# Protective Action of Resveratrol in Human Skin: Possible Involvement of Specific Receptor Binding Sites

**DOI:** 10.1371/journal.pone.0012935

**Published:** 2010-09-23

**Authors:** Stéphane Bastianetto, Yvan Dumont, Albert Duranton, Freya Vercauteren, Lionel Breton, Rémi Quirion

**Affiliations:** 1 Department of Psychiatry, Douglas Mental Health University Institute, McGill University, Montréal, Quebec, Canada; 2 L'Oréal Recherche, Clichy, France; Hungarian Academy of Sciences, Hungary

## Abstract

**Background:**

Resveratrol is a plant-derived polyphenol with purported protecting action on various disorders associated with aging. It has been suggested that resveratrol could exert its protective action by acting on specific plasma membrane polyphenol binding sites (Han Y.S., et al. (2006) J Pharmacol Exp Ther 318:238–245). The purpose of this study was to investigate, in human skin, the possible existence of specific binding sites that mediate the protective action of resveratrol.

**Methods and Findings:**

Using human skin tissue, we report here the presence of specific [^3^H]-resveratrol binding sites (K_D_  = 180 nM) that are mainly located in the epidermis. Exposure of HaCaT cells to the nitric oxide free radical donor sodium nitroprusside (SNP; 0.3–3 mM) resulted in cell death which was reduced by resveratrol (EC_50_  = 14.7 µM), and to a much lesser extent by the resveratrol analogue piceatannol (EC_50_  = 95 µM) and epigallocatechin gallate (EC_50_  = 200 µM), a green-tea derived polyphenol. The protective action of resveratrol likely relates to its anti-apoptotic effect since at the same range of concentration it was able to reduce both the number of apoptotic cells as well as mitochondrial apoptotic events triggered by SNP.

**Conclusion:**

Taken together, these findings suggest that resveratrol, by acting on specific polyphenol binding sites in epidermis, may be useful to prevent skin disorders associated with aging.

## Introduction

Resveratrol (3,5,4′-trihydroxystilbene) is a naturally occurring polyphenol and is particularly found in grape skin, nuts and pomegranate. It has been hypothesized that resveratrol contributes to the ability of polyphenols rich Mediterranean diet to reduce the incidence of age-related diseases such as coronary heart disease [1], [2], cancer [3] and dementia [4]. In support of this hypothesis, resveratrol displays a broad variety of beneficial effects including cardioprotective, neuroprotective, antimicrobial and chemopreventive properties [5]–[9]. Resveratrol, for example has been shown to down-regulate vasoactive peptides such as endothelins [10], to inhibit oxidized low-density lipoprotein [11] and cyclooxygenase [2], to inhibit the clearance and neurotoxicity of beta-amyloid (Aß) [12], [13], to modulate apoptotic signalling pathways [8] and to activate sirtuin and AMP-activated protein kinase which are believed to be involved in the caloric restriction-longevity effect [14], [15]. We have previously reported that resveratrol shared with other polyphenols (e.g. quercetin and catechins), the ability to block neuronal hippocampal cell death against the toxicity induced by nitric oxide (NO) [16] and Aß peptides [13], raising the possibility for polyphenols to be beneficial in the prevention of age-related neurodegenerative disorders [17]–[20]. Membrane binding and autoradiographic studies on rat brain suggest the existence of polyphenol specific plasma membrane binding sites, possibly underlying the neuroprotective action of polyphenols [21].

The characterization of resveratrol as a promising agent with anti-aging properties, in addition to many other health benefits, has prompted interest in investigating its protective effects against factors known to alter the course of normal skin aging [22]–[24]. Here, we hypothesize that resveratrol could exert its protective actions through specific binding sites located in the epidermis. Accordingly, the main objective of the present study was to identify and characterize the presence of specific binding sites for [^3^H]-resveratrol in human skin tissue. Abnormal increase in NO release has been reported to be involved in premature skin aging, in part via its stimulatory effects on reactive oxygen species and apoptotic pathways [25]–[28]. Using human keratinocytes cell line (HaCaT), we investigated the protective effects of resveratrol against cell death induced by sodium nitroprusside (SNP), a well known NO releasing with purported toxic and apoptotic effects in keratinocytes [29].

## Results

### [^3^H]-Resveratrol binding sites in human skin sections and in HaCaT cells

**Figure 1:**
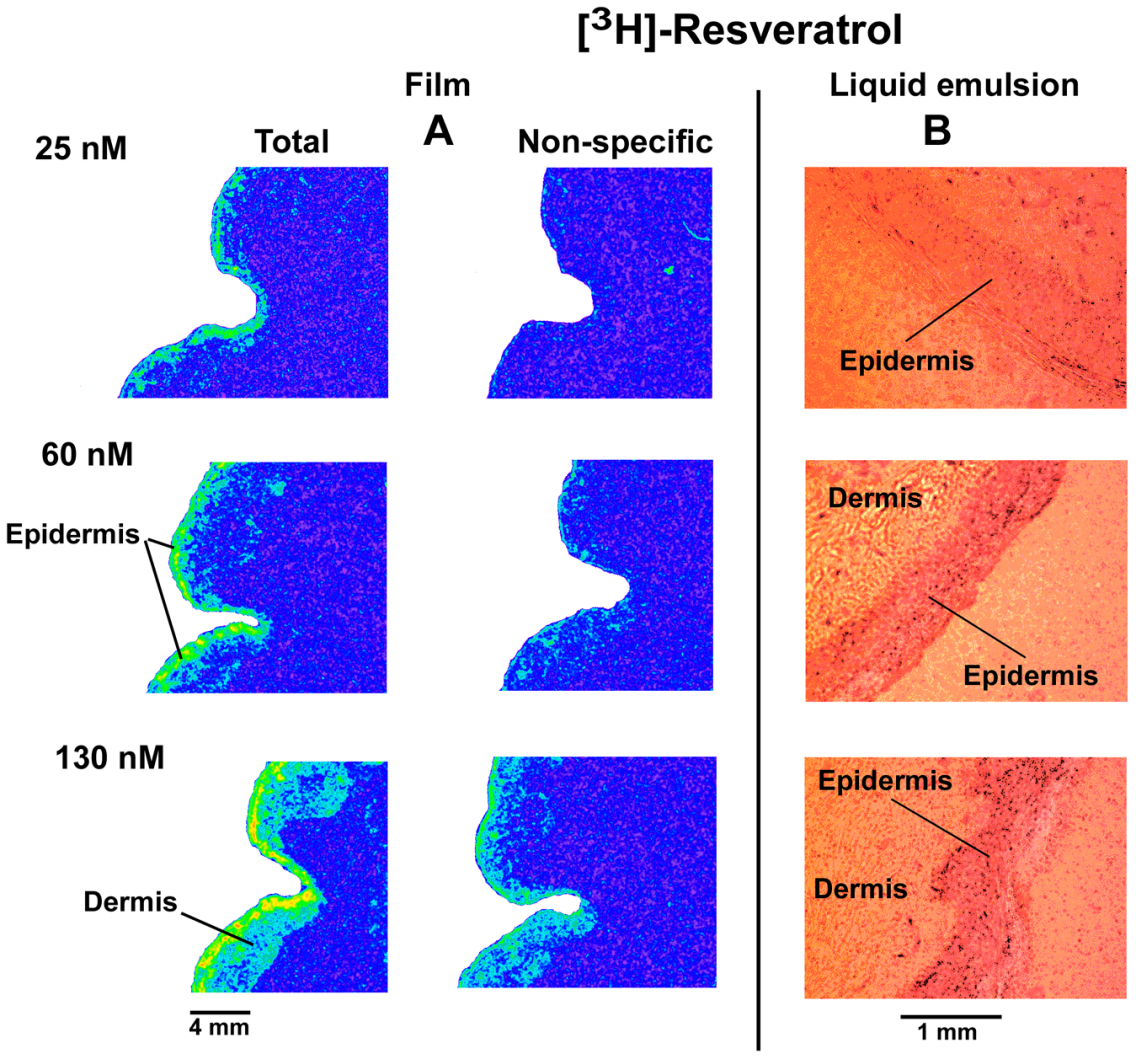
Photomicrographs of the autoradiographic distribution of [^3^H]-resveratrol binding sites in human skin. Upper panel (A). Human skin sections were incubated during 8 months with [^3^H]-resveratrol (25, 60 and 130 nM), stained with hematoxylin/eosin and visualized using a microscope. Higher levels of silver grains were observed in the epidermis as compared to the dermis. Total and non-specific binding represents [^3^H]-resveratrol binding with or without cold resveratrol (100 µM), respectively. Lower panel (B). Photomicrographic distribution of human skin incubated with [^3^H]-resveratrol (25, 60 and 130 nM) and exposed to liquid emulsion for 8 months. The widespread distribution of silver grains (black dots) in the epidermis suggested that [^3^H]-resveratrol binding sites are mostly located in keratinocytes, which makes about 90% of skin epidermis. The number of silver grains is similar in undifferentiated (cells in the basal layer) and differentiated (cells at the surface of the skin) keratinocytes.

**Figure 2:**
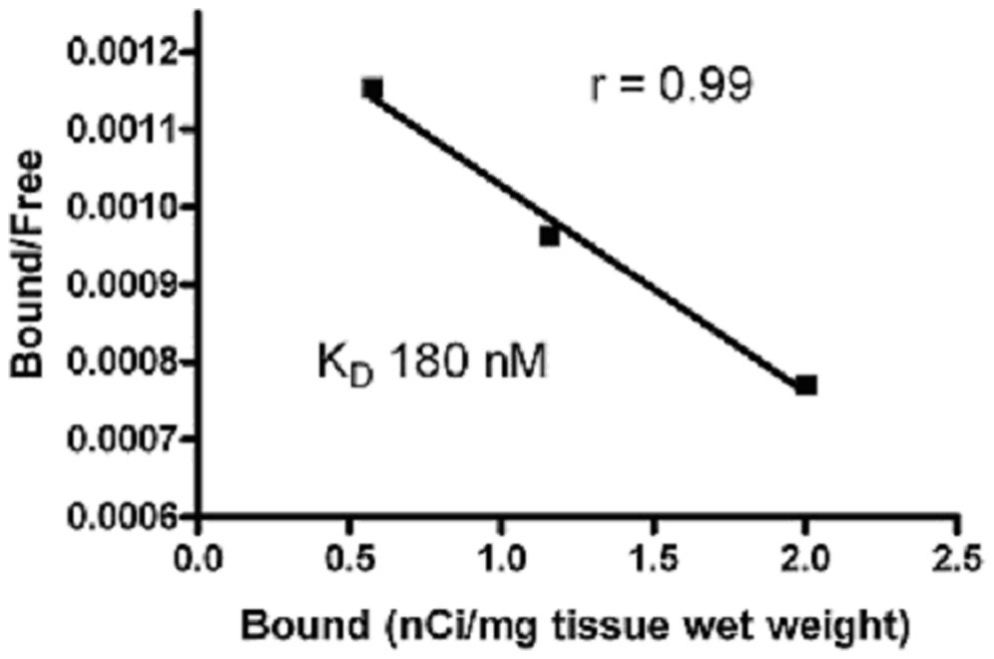
Scatchard transformation of data presented in **Table 1**. The affinity (KD) and maximal binding capacity (Bmax) of the saturation isotherms were estimated by Scatchard transformation of 12 sections from 3 human skins.

**Figure 3:**
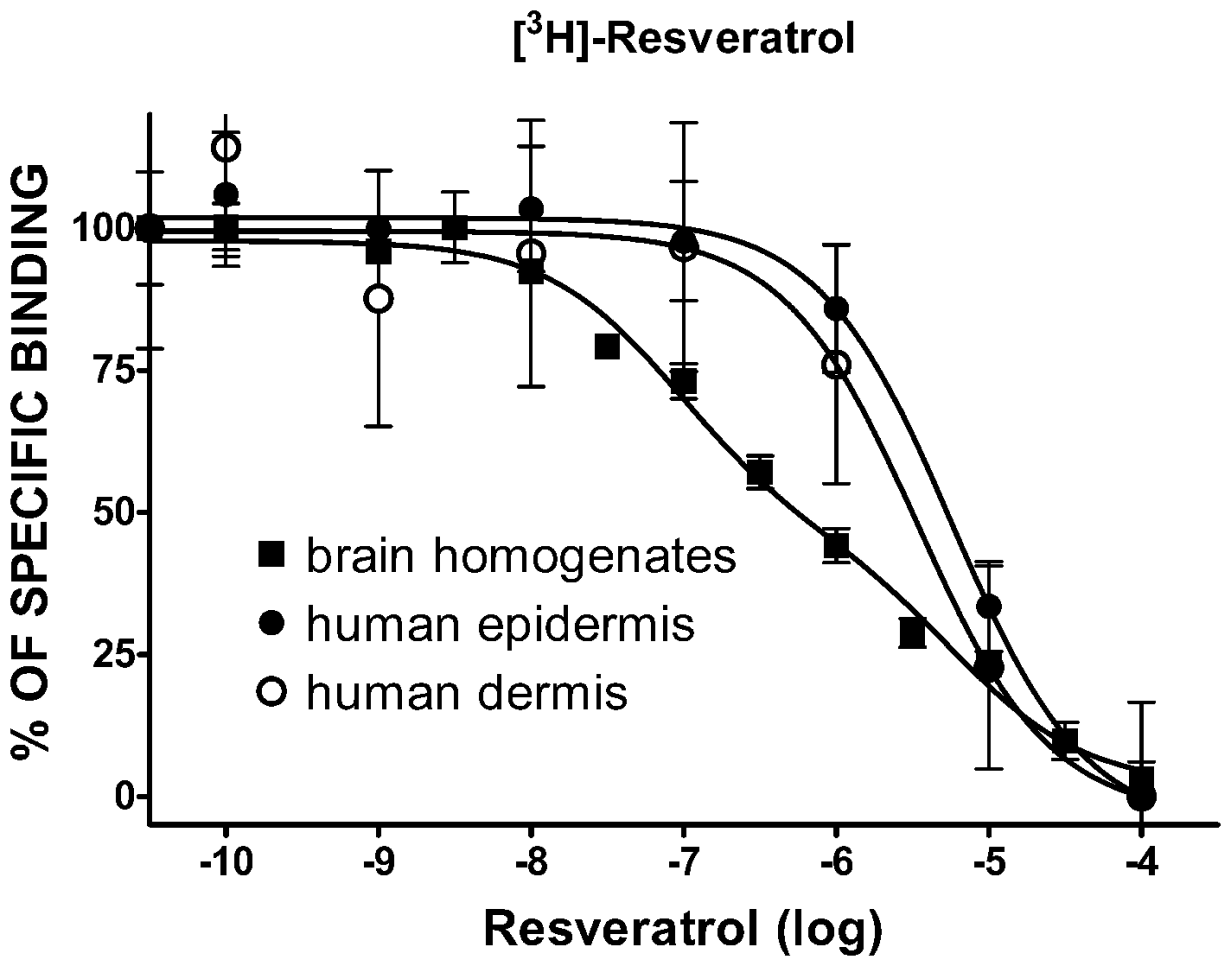
Competition binding profile of resveratrol against [^3^H]-resveratrol binding site in human skin and brain homogenates. Brain homogenates and human skin sections were incubated with [^3^H]-resveratrol in the presence of increasing concentration of cold resveratrol (0.1 nM–100 µM). Each point represents the mean±SEM of data obtained from three to five determinations, each performed in triplicate and expressed as percentage of specific binding.

**Figure 4:**
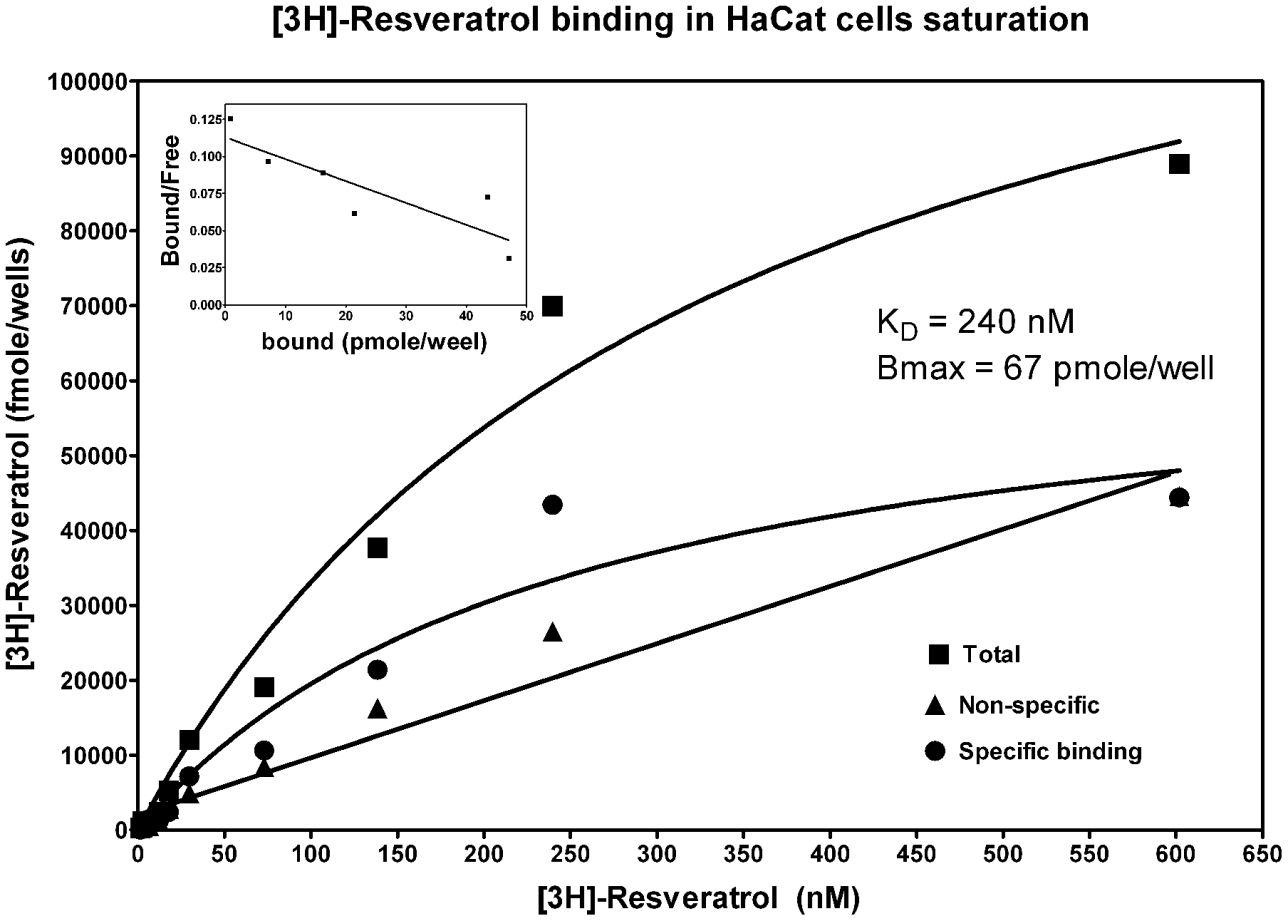
Typical profile of saturation binding isotherms of [^3^H]-resveratrol in HaCaT cells. Binding experiments were performed at 4°C in the presence or the absence of 100 µM unlabelled resveratrol; specific (round points) binding represents the difference between total (square points) and non-specific (triangle points) binding. Insert shows the mathematical transformation as Scatchard plots of the binding isotherms of [^3^H]-resveratrol. Each point was done in triplicate and repeated 3 times.

We aim to determine first the presence and distribution of [^3^H]-resveratrol binding sites using autoradiography. As shown in Figure 1A, significant amount of specific [^3^H]-resveratrol labeling was observed in the epidermis and to a much lesser extent in the dermis. The intensity of the signal obtained with [^3^H]-resveratrol was dependent on the concentration of radioligand used (Fig. 1A). Similarly, the differential distribution of [^3^H]-resveratrol binding sites in human sections exposed to liquid emulsion indicated that most of the [^3^H]-resveratrol binding sites are found in granular keratinocytes, which represent more than 90% of cells in the epidermis (Fig. 1B). Quantification of [^3^H]-resveratrol binding obtained from human skin sections confirmed that the epidermis contained much higher amounts of specific [^3^H]-resveratrol binding sites as compared to the dermis (Table 1) with an apparent affinity (K_D_) of 180±50 nM (Fig. 2). Additionally, competition binding experiments on human skin sections revealed that resveratrol competed for 50% of specific [^3^H]-resveratrol (30 nM) binding sites with a Ki value of 2.6±0.7 and 1.5±0.6 µM in the epidermis and dermis, respectively, which are highly similar to low affinity sites observed in brain homogenates (Fig. 3; see also [21]). Moreover, the presence of resveratrol binding sites has been also observed in human immortalized keratinocyte HaCaT cells, and isotherm saturation binding experiments revealed the presence of high affinity sites (K_D_ of 240 nM; Fig. 4). These data confirm the presence of [^3^H]-resveratrol binding sites in keratinocyte cells and has been further used to investigate the *in vitro* protective effects of resveratrol against SNP-induced toxicity.

**Table 1:** Quantitative distribution of [^3^H]-resveratrol binding in human skin.

[^3^H]-Resveratrol	Specific binding sites (nCi/mg tissue wet weight)	
	Epidermis	Dermis
25 nM	0.577±0.04	0.009±0.011
60 nM	1.156±0.09	0.066±0.06
130 nM	2.003±0.15	0.133±0.08

Levels of [^3^H]-resveratrol binding were quantified using a MCID image analysis (St. Catherines, ON, Canada). Total [^3^H]-resveratrol binding was subtracted from adjacent sections incubated in the presence of 100 µM unlabelled resveratrol and expressed as specific [^3^H]-resveratrol binding sites (nCi/mg tissue, wet weight). Data represent the mean ± SEM of 12 sections obtained from 3 different subjects.

### Resveratrol protects HaCaT cells against SNP-induced toxicity

**Figure 5:**
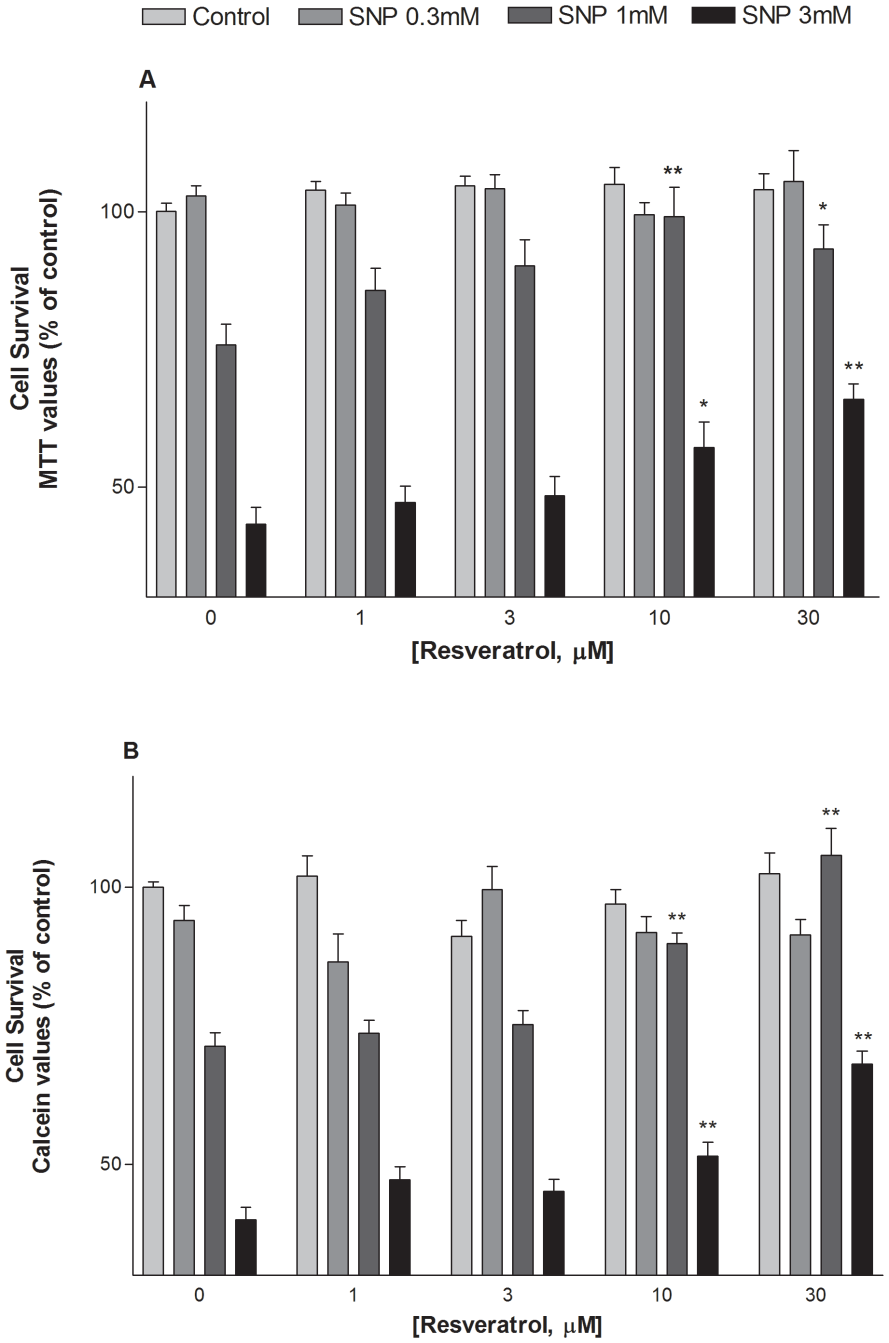
Effect of resveratrol against cell death induced by SNP in HaCaT cells. Cells were exposed to SNP (0.3–3 mM) in the presence or absence of resveratrol (1–30 µM). Cell viability was determined 24 hours later using both the MTT and calcein AM assays. Values represent mean ± SEM of at least three separate experiments. *p<0.05, **p<0.01 comp.

We then performed a functional assay to evaluate the capacity of resveratrol to protect HaCaT cells against the toxicity induced by the NO releasing SNP. Treatment of HaCaT cells with SNP (0.3–3 mM) inhibited the growth of HaCaT cells in a concentration dependent manner, as evaluated by the MTT and calcein assays (Fig. 5*A, B*). Both assays showed that resveratrol (1–30 µM) strongly attenuated SNP-induced toxicity, producing a significant effect at 10 µM and a maximal one at the highest concentration tested (Fig. 5*A, B*).

**Figure 6:**
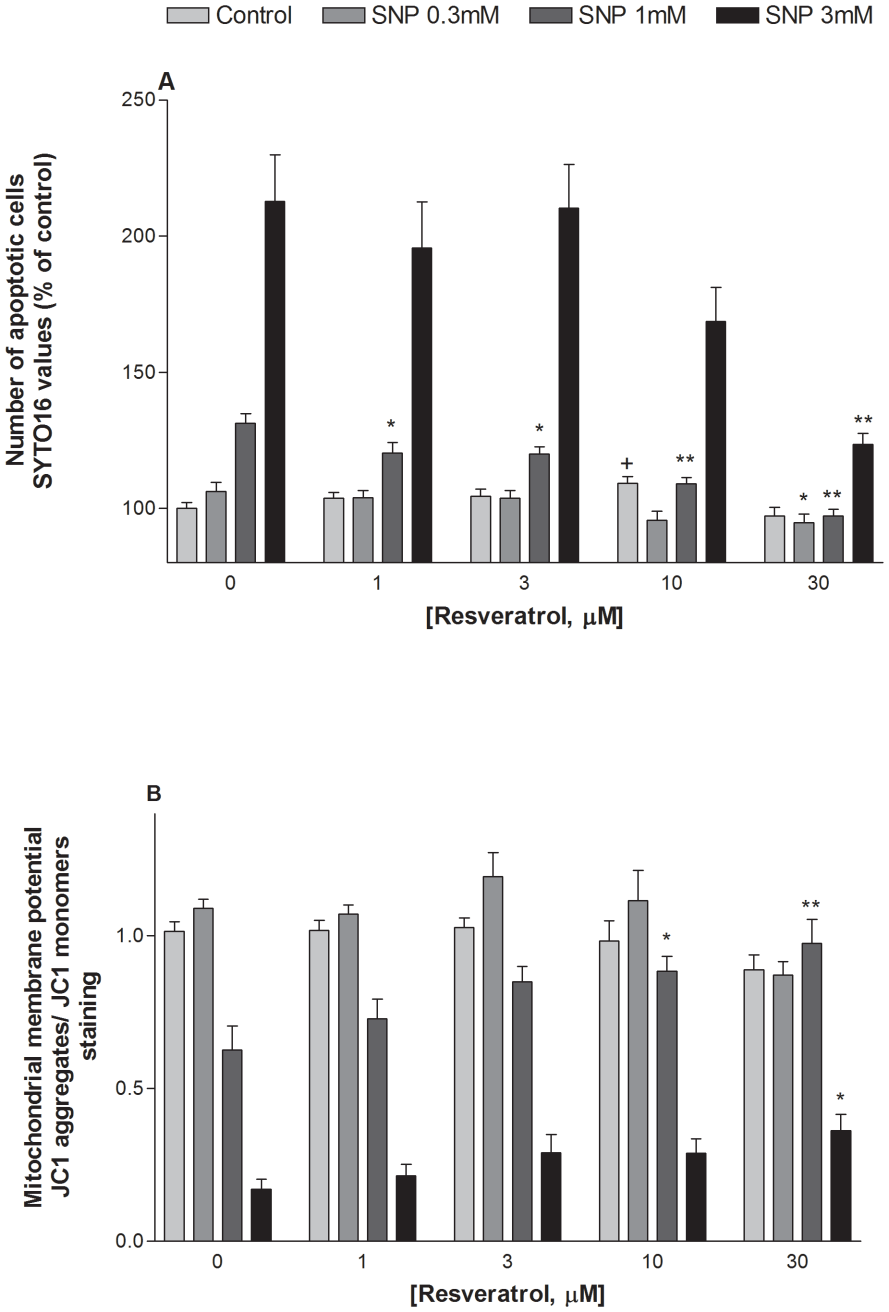
Effect of resveratrol against apoptotic events induced by SNP in HaCaT cells. Cells were exposed to SNP (0.3–3 mM) in the presence or absence of resveratrol (1–30 µM). Cell viability was determined 24 hours later using both the SYTO 16 (A) and JC-1 (B) assays. Values represent mean ± SEM of at least three separate experiments. *p<0.05, **p<0.01 compared to groups treated with SNP alone. +p<0.05 compared to control group.

The number of SNP-treated cells stained with SYTO 16 increased, indicating that SNP exerted an apoptotic effect on HaCaT cells (Fig. 6*A*). The increase in apoptotic cells was reduced when treated with resveratrol (1–30 µM) with a significant inhibitory effect at 1 µM (Fig. 6*A*). The JC1 assay confirmed the apoptotic effect of SNP resulting in the loss of mitochondrial membrane potential (MMP), as indicated by a decrease in the red/green fluorescence intensity ratio (Fig. 6*B*). Resveratrol reversed the loss of MMP, with significant effect at the concentrations with an anti-apoptotic effect (Fig. 6*B*).

**Figure 7:**
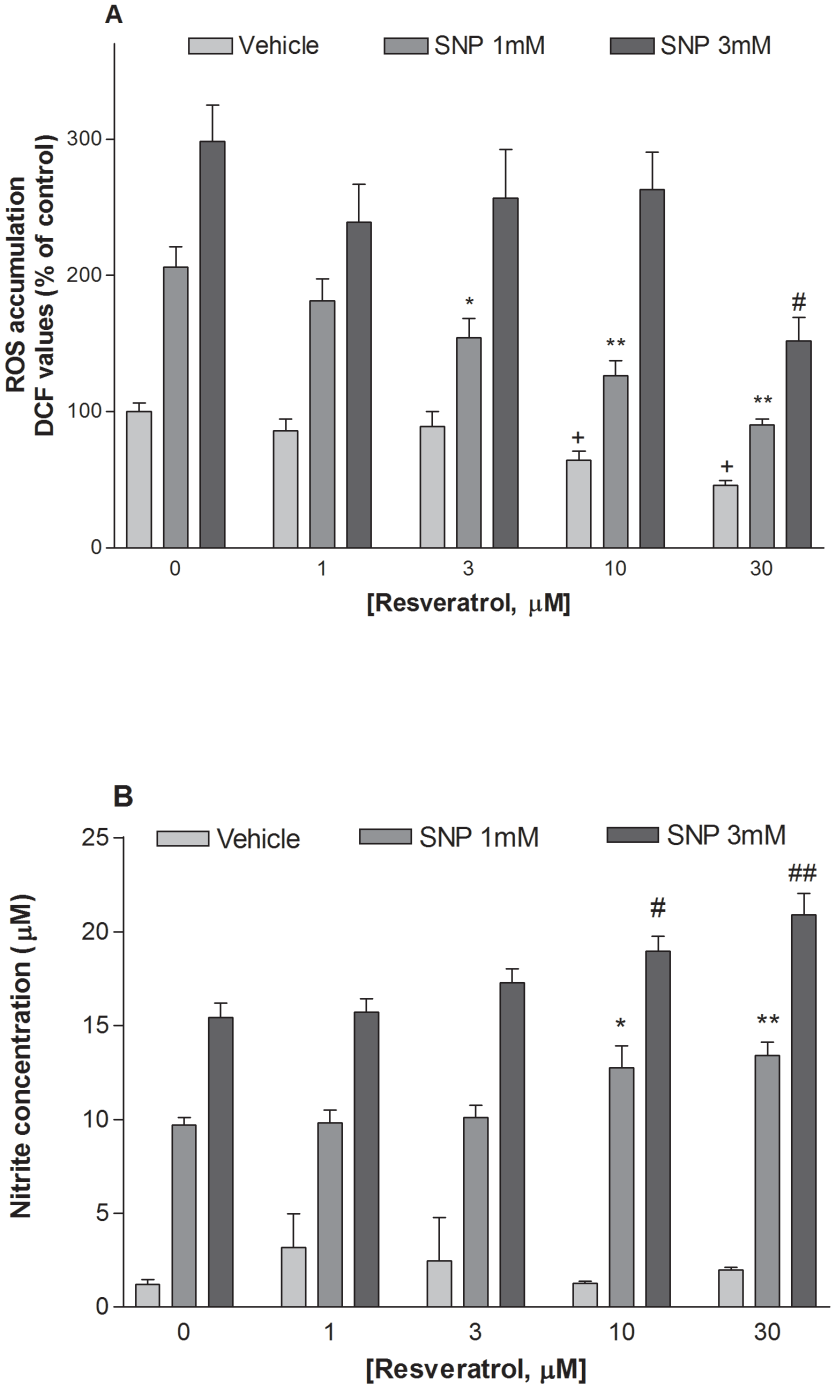
Effect of resveratrol against ROS (A) and nitrite (B) accumulation induced by SNP in HaCaT cells. Cells were exposed to SNP (1 and 3 mM) in the presence or absence of resveratrol (1–30 µM). ROS and nitrite accumulation was determined 5 hours later using the DCF assay and the Griess reagent respectively. Values represent mean ± SEM of at least four separate experiments. *p<0.05, **p<0.01 compared to groups treated with 1 mM SNP only. #p<0.05, ##p<0.01 compared to groups treated with 3 mM SNP alone. +p<0.05 compared to control group.

The DCF assay indicated that a 5-hour exposure to SNP (1 and 3 mM) causes a significant increase (*P<0.01) in ROS accumulation (106 and 198% above control values, respectively; Fig.7*A*). SNP-stimulated ROS production was significantly attenuated by resveratrol (1–30 µM) in the range of concentrations that protected hippocampal cells against SNP-induced toxicity (Fig. **7**A). The effect of resveratrol was significant at 3 µM (vs SNP 1 mM) and 30 µM (vs SNP 3 mM), whereas ROS accumulation in cell cultures treated with resveratrol (10–30 µM) alone was slightly, but significantly, decreased compared to vehicle-treated control groups (Fig. 7*A*). Additionally, a 5-hour exposure to SNP (1–3 mM) increased nitrite level in the culture medium (P<0.01). However, resveratrol (1–30 µM) failed to attenuate NO production at the concentrations that protected HaCaT cells against SNP (Fig. 7*B*).

### Comparative effects of polyphenols against SNP-induced cell death and ROS accumulation

**Figure 8:**
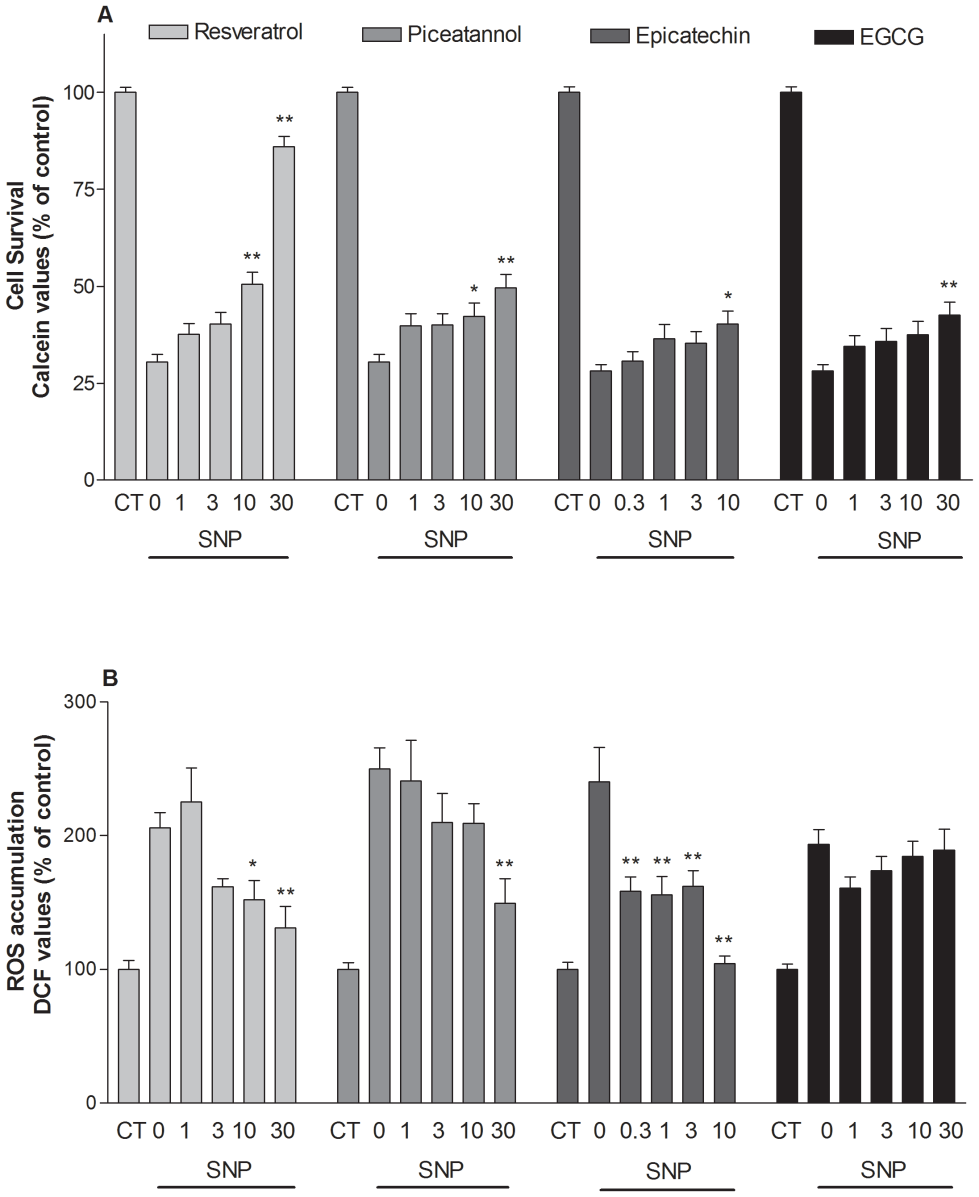
Comparison of polyphenols against cell death (A) and ROS accumulation (B) induced by SNP in HaCaT cells. Cells were exposed to SNP (2 mM) in the presence or absence of polyphenols (0.3–30 µM). ROS accumulation was determined 5 hours later using the DCF assay. Values represent mean ± SEM of at least three separate experiments. *p<0.05, **p<0.01 compared to groups treated with 2 mM SNP alone.

We compared the antioxidant properties of various polyphenols [(i.e. resveratrol and its analog piceatannol, epicatechin and its gallate ester epigallocatechin gallate (EGCG)] and their capacity to block the toxicity of SNP in HaCaT cells, using the DCF and calcein assays, respectively. Among the polyphenols tested here, resveratrol was most effective to protect cells against SNP-induced toxicity with an EC_50_ of 14.7 µM, followed by epicatechin (EC_50_ = 58.9 µM), while piceatannol and epigallocatechin gallate (EGCG) were less effective (EC_50_ = 94 and 140 µM, respectively) (Fig. 8*A*). Epicatechin was the most potent to inhibit ROS with an EC_50_ value of 0.11 µM followed by resveratrol (EC_50_ = 5.9 µM) and piceatannol (EC_50_ = 29.4 µM), while EGCG failed to show antioxidant properties (EC_50_>100 µM) (Fig. 8*B*). The antioxidant activity of resveratrol and other polyphenols did not correlate (r = 0.16) with their protective activity against SNP-induced toxicity, suggesting that the protective effects exhibited by resveratrol and congeners are not associated with their ability to block ROS accumulation generated by SNP.

### Resveratrol inhibits SNP-induced both caspases 3 and 9 activities induced by SNP, but the p38MAPK pathway in theses processes

**Figure 9:**
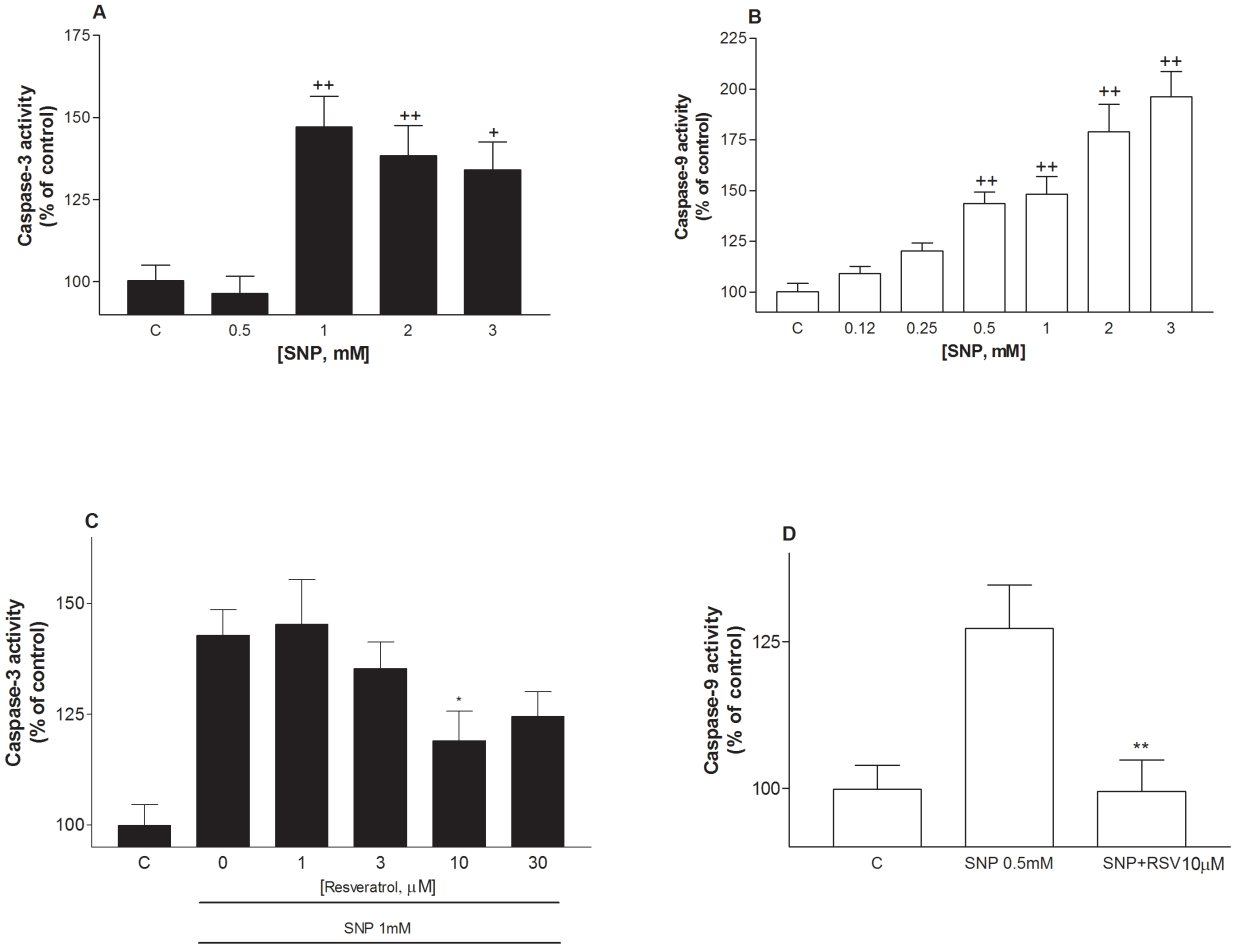
Effect of resveratrol against SNP-induced caspase-3 and caspase-9 activation in HaCaT cells. Cells were exposed to SNP (0.12–3 mM) in the presence or absence of resveratrol (1–30 µM). Caspase-3 and -9 activities were measured 24 hours later using colorimetric assay kits. Values represent mean ± SEM of at least three separate experiments. +p<0.05, ++p<0.01 compared to control group. *p<0.05, **p<0.01 compared to groups treated with SNP alone.

ELISA assays were performed to investigate whether the neuroprotective action of resveratrol involved a modulation of the activity of caspases and p38 MAPK, two intracellular effectors that have been reported to be involved in the apoptotic effect of NO production (30). The caspase-3 inhibitor (5 µM) significantly increased cell viability compared to 2 mM SNP-treated cells [59.5±5 vs 26.2±2; p<0.01 (calcein values)], whereas the caspase-9 inhibitor (5 µM) was not effective [36.9±2 vs 32±2; NS (calcein values)]. A role for caspases in NO-induced apoptosis in HaCaT cells was confirmed by ELISA as SNP (0.5–3 mM) increased the activity of both caspases-3 and -9 with a significant effect at 1 mM and 0.5 mM, respectively (Fig. 9*A,B*). In a concentration-dependent manner resveratrol reduced the increase in activity of caspase-3 with a significant effect at 10 µM (Fig. 9*C*). Moreover, resveratrol was able to completely block SNP-induced caspase-9 activity (Fig. 9*D*). Co-exposure of 2 mM SNP with either SB 203580 (25 µM) or SB 202190 (25 µM), two inhibitors of p38 MAP kinase [30], resulted in an increase in cell viability (Table 2). However, resveratrol failed to regulate either form of p38 MAPK at concentrations that protected HaCaT cells (Table 2).

**Table 2:** Effect of resveratrol and SB 203580 on p38 MAPK levels induced by SNP in HaCaT cells.

Treatment	Relative level ofPhospho-p38 MAPK(% of control)	Relative level oftotal p38 MAPK(% of control)
Control	100±1	100±2
SNP (2 mM)	125±3	118±2
+ Resveratrol (20 µM)	124 ± 4	120±3
Control	100±1	100±2
SNP (2 mM)	119±3	119±3
+ SB 203580 (25 µM)	105 ± 3*	102±3*

Values represent mean ± SEM of four separate experiments.

*p<0.01 compared to groups treated with SNP alone.

### Microarray analysis of genes related to NO signalling pathway

A quantitative real-time RT-PCR was performed to evaluate if resveratrol was able to modulate the expression of various genes known to be involved in NO signalling pathway. Among all the genes investigated here, those that display fold change higher than threshold are indicated in Table S1. HaCaT cells treated with SNP (1 mM) show that 10 genes were upregulated while 6 genes were down regulated. Resveratrol (10 µM) was able to restore the expression of SNP up-regulated (e.g. interleukin 8, jun oncogene, nitric oxide synthase 3, NADPH dehydrogenase, quinone 1) or down-regulated (e.g. catalase, glutathione peroxidase) genes. Moreover, resveratrol was independently able to increase gene expression including glutathione peroxidase, keratin 1 and oxidation resistance 1 and decrease the expression of ribosomal protein L13a (Table S1).

## Discussion

In the present study we describe for the first time, the existence of [^3^H]-resveratrol specific binding in human skin and human keratinocytes cell line (HaCaT). Receptor autoradiography revealed that specific [^3^H]-resveratrol binding is present in human skin tissue with the highest level of labelling seen in the epidermis and apparent affinity (K_D_) of 180±50 nM. The presence of resveratrol binding sites has been also observed in HaCaT cells, and isotherm saturation binding experiments revealed the presence of high affinity sites (K_D_ of 240 nM). Interestingly, we have previously demonstrated the existence of a high affinity polyphenols binding in the brain, with an apparent affinity (K_D_) of 220 nM [21], which is quite similar to that reported here in both human skin tissue and HaCaT cells. Furthermore, competition binding experiments have shown that resveratrol was able to inhibit specific [^3^H]-resveratrol binding sites in the epidermal and dermal layers with Ki values of 2.6 and 1.5 µM, respectively, which are highly similar to low affinity sites observed in brain homogenates (present data manuscript; see also [21]. However, the fact that we could not detect a significant proportion of binding in the high affinity state in the human skin suggest that binding sites in high affinity state only represent a small proportion of total binding in the human skin. Further investigation will be required to identify the nature of resveratrol binding sites observed in human skin.

Numerous studies have demonstrated that resveratrol show potent protective effects in various models of toxicity [13], [16], [20] including toxicity induced by NO releasing SNP [16]. Similarly, resveratrol protected HaCaT cells exposed to SNP, suggesting its ability to block the deleterious events induced by NO overproduction, a process relevant to premature skin aging occurring upon long term UV exposure [25]. We compared the antioxidant properties of various polyphenols and their capacity to block the toxicity of SNP in HaCaT cells. Among the polyphenols tested here, resveratrol was the most effective to protect cells against SNP-induced toxicity with an EC_50_ of 14.7 µM followed by epicatechin (EC_50_ = 58.9 µM), while piceatannol and EGCG were less effective (EC_50_ = 94 and 140 µM, respectively). This is in agreement with previous studies reporting a protective action of resveratrol and EGCG against SNP-induced toxicity at the same range of concentration [31]. Furthermore, competition binding experiments have shown that resveratrol was the most potent competitor in HaCaT cells withµmolar affinity (resveratrol > piceatannol > EGCG) (data not shown). Several studies have shown that NO-induced toxicity is engendered by its reaction with the superoxide anion radical (O2−) yielding highly cytotoxic reactive oxygen species (ROS), peroxynitrite. This molecule is degraded to produce nitrogen dioxide and the radical hydroxyl, leading to DNA damage [32]. However, the purported antioxidant activities and ROS scavenging properties of resveratrol do not fully explain its protective capacity. Indeed, the antioxidant properties of the polyphenols tested here did not correlate to their ability to protect HaCaT cells against SNP-induced toxicity. Moreover, resveratrol was unable to modulate the activity of nitric oxide synthase (NOS) elicited by SNP (as measured by nitrite accumulation), indicating that its protective action is not likely to be directly associated to the inhibition of NOS. This is in agreement with our previous report showing that resveratrol (10 µM) failed to inhibit the production of NO in hippocampal cells [16]. Taken together, this suggests that the protective effect of resveratrol observed in the present model does not involve its ROS scavenging properties but rather a modulation of intracellular effectors.

In addition to their antioxidant properties, polyphenols such as resveratrol have been reported to modulate the properties of markers of apoptosis [33], [34] suggested to be activated during NO-mediated cell injury [25]. Accordingly, SNP increased the activity of the apoptotic markers such as caspases 3 and 9, which was significantly reduced in the presence of resveratrol at the same concentration range showing a protective action. These data agree with previous findings reporting that the polyphenol 3,4′-DHF was found to exert an anti-apoptotic effect on etoposide-induced cell death of HaCaT cells [35]. This also implies that the inhibitory effect of resveratrol on increased activity of caspase-3 - a critical effector of mitochondrial events in apoptosis [26] could explain at least in part, its protective action against the toxic effect of SNP. In support of this hypothesis, we report here that resveratrol reduced the increase in number of apoptotic cells and also reversed the loss of mitochondrial membrane potential initiated by SNP. Moreover, treatment with a caspase-3 inhibitor significantly increased cell viability, while an inhibitor of caspase-9 was ineffective, indicating that the activation of caspase-3 (a caspase effector) is a crucial step leading to the apoptotic cell death induced by SNP.

It has been reported that p38 MAPK is one of the most prominent protein kinase involved in NO-induced apoptosis upstream of caspase activation [30]. In support of this hypothesis, we show that (1) SNP-triggered cell death was reduced in the presence of two inhibitors of p38 MAPK (i.e. SB 203580 and SB 202190) [30] and (2) SNP activated both phospho-p38 and the total level of p38 MAPK. However, resveratrol failed to regulate either form of p38 MAPK at concentrations that protected HaCaT cells, suggesting that the protective action of resveratrol did not involve this protein kinase. The lack of additive effects of resveratrol and p38 MAP kinase inhibitors in protecting HaCaT cells against SNP-induced cell death suggests that inhibition of caspase-3 by resveratrol depends on p38 MAP kinase activity. It thus appears that p38 MAP kinase is not directly involved in the mitochondrial injury induced by SNP.

Finally, results from quantitative RT-PCR indicate that resveratrol (10 µM) reversed SNP-altered expression of genes that either protect cells against oxidative stress (e.g. catalase, glutathione peroxidase) or promote oxidative stress (e.g. NADPH dehydrogenase quinone 1) and inflammation (e.g. interleukin 8 and NOS). Moreover, resveratrol by itself upregulated the expression of genes that protect against oxidative damage (i.e. glutathione peroxidase, oxidation resistance 1) or were involved in keratinocyte differentiation [i.e. keratin 1, [36]] and cutaneous wound healing [(i.e. vascular endothelial growth factor A, [37]].

In summary, here we show that resveratrol possibly exerts its protective effect on epidermal cells by binding on specific polyphenol binding sites. Intracellular mechanisms underlying the protective effect of resveratrol are not solely due to its antioxidant activities but also involve its inhibitory action on apoptosis, a downstream mitochondrial dysfunction, as revealed by the ability of resveratrol to reduce caspase-3 activity as well as the number of apoptotic HaCaT cells and to prevent mitochondrial dysfunctions. It will be of interest to confirm these results using primary keratinocytes from human samples. Considering that NO is a key mediator implicated in a broad range of age-related skin damages, these findings suggest that resveratrol could delay and even prevent the normal course of skin aging by blocking apoptotic events and mitochondrial dysfunctions. These data also suggest that it could be possible to develop analogues of resveratrol with a higher affinity towards polyphenols binding sites, leading to pertinent effects in reducing skin aging.

## Materials and Methods

### Materials

Human immortalized HaCaT keratinocytes cells were purchased from CLS Cell Lines Service (Eppelheim, Germany). Materials used for cell cultures were obtained from Invitrogen (Burlington, ON, Canada). Fluorescent dyes (calcein, SYTO16 green, 2′,7′-dichlorofluorescein diacetate, dye 5,5′,6,6′-tetrachloro-1,1′,3,3′ tetraethylbenzimidazolylcarbocyanine iodide) and Griess reagent kit were purchased from Invitrogen-Molecular probes (Burlington, ON, Canada ). Caspases-3 and -9 inhibitors were purchased from Calbiochem (La Jolla, CA, USA). Caspases-3 and -9 colorimetric assay kits were obtained from Chemicon International (Temecula, CA, U.S.A.), whereas the RayBio® Cell-Based p38 MAPK (Thr180/Tyr182) ELISA Kit was from RayBiotech (Norcross, GA, U.S.A.). [^3^H]-Resveratrol (specific activity 0.3 to 0.6 Ci/mmole) was purchased from Moravek Biochemicals Inc. (Brea, CA, USA). Materials for quantitative real-time RT-PCR including the human NO signalling pathway RT^2^ profiler PCR array were purchased from SABiosciences (Frederick, MD, U.S.A.). Unless otherwise stated, all other compounds were purchased from Sigma-Aldrich (St. Louis, MO, U.S.A.). Stock solutions (10^−1^M) of resveratrol and other drugs were dissolved in dimethylsulfoxide (DMSO). This solvent at 0.03% (v/v) has no effect by itself on cell survival (data not shown).

### Human skin tissue

Human skin tissue was obtained from patients (female; n = 3; 50, 60 and 65 years old) who were going to have plastic surgery (lifting). Before surgical operation, patients were asked for their consent, if the tissue removed during operation could be used for resveratrol research studies. All human skin tissues used in this study were from patients who had given their written consent. Additionally, the study protocol and the consent form were approved by the Douglas Institute Ethic committee. Informed written consent of patients was obtained before tissue resection and the study was conducted according to the Declaration of Helsinki Principles.

### [^3^H]-Resveratrol binding on human skin sections

Immediately after surgical removal, skin tissues were kept in humidified (0.9% NaCl solution) pad at 4°C and transported to the Douglas Mental Health University Institute. Tissues were immediately frozen in 2-methylbutane at −40°C for 15 sec and then kept at −80°C until needed. Human skin sections (16 µm) were obtained using a cryomicrotome at −17°C, mounted on Fisher Superfrost slides, dried overnight in a desiccator at 4°C, and then kept at −80°C until use. Receptor autoradiography was performed as described in detail elsewhere [21]. Briefly, human skin sections were incubated in the presence of [^3^H]-resveratrol at concentration of 25, 60 and 130 nM. Levels of [^3^H]-resveratrol binding were quantified using a MCID image analysis (St. Catherines, ON, Canada) Total [^3^H]-resveratrol binding was substracted from adjacent sections incubated in the presence of 100 µM unlabelled resveratrol and expressed as specific [^3^H]-resveratrol binding (nCi/mg tissue, wet weight). Competition binding assays were done at 30 nM of [^3^H]-resveratrol in the presence of increasing concentration of cold resveratrol ranging from 0.1 nM to 100 µM.

At the end of the incubation period, human skin sections were washed 4 times, one min each in cold Krebs-Ringer phosphate buffer (KRP), rapidly dipped in cold water, fixed in 2.5% formalin and 2.5% glutaraldehyde in phosphate buffered saline (PBS) for 10 min, washed in PBS and dehydrated in successive baths of 70%, 90% and 100% ethanol (5 min each). Sections were apposed against Kodak Biomax MR films (PerkinElmer, Woodbridge, ON, Canada) or liquid emulsion type NTB (Eastman Kodak, Rochester, NY, USA) for eight months alongside with [^3^H]-radioactive standards (GE Healthcare Canada, Baie d'Urfe, QC, Canada).

### Cell culture

HaCaT cells were prepared as described previously, with minor modifications [38]. Briefly, HaCaT cells were cultured in HEPES-buffered Dulbecco's modified Eagle medium (DMEM) high glucose, 10% fetal bovine serum (FBS) and 1% penicillin/streptomycin. Cells were grown by removing the original medium twice a week and replacing it with the medium of the same composition until they reached confluence. Cells were then removed from 75 cm^3^ culture flasks by trypsinization (0.25% trypsin in 0.02% EDTA-PBS) and seeded in either 6 well or 96 well plates (Corning Inc., Corning, NY, USA). Cultures were maintained at 37°C in a humidified atmosphere (5% CO_2_ and 95% air) and used for experiments once they reached confluence.

### [^3^H]-Resveratrol binding on HaCaT cells

Binding assays were performed in HaCaT cells, once reached to confluence in 6-well plates and each point was done in triplicate. Cells were washed twice with cold KRP buffer and binding assays were performed at 4°C by adding 2.5 ml of KRP buffer containing [^3^H]-resveratrol (0.5 to 600 nM) in the presence and absence of 100 µM cold resveratrol (non-specific binding). The binding reaction was stopped by two rapid washes of 5 ml cold Krebs buffer. Cells were lysed with 0.5 ml of 1N NaOH containing 1% TritonX100 for 30 min and neutralized with 0.5 ml of 1N HCl. Radioactivity was quantified using a beta counter with 45% efficiency (Beckman Instruments, Meriden, CT, USA).

### Calcein and MTT assays

SNP-induced toxicity was performed in HaCaT cells plated in 96 wells. On the day of the experiment, the medium was removed and replaced with similar medium with SNP (0.3–3.0 mM) and without FBS in the presence or absence of different drugs. Cell viability was determined 24 hours later using both the calcein AM and the 3-(4,5-dimethylthiazol-2-yl)-2,5-diphenyl tetrazolium bromide (MTT) assays, as described previously [16], [39].

### SYTO 16 staining of apoptotic nuclei

Nuclear staining was performed using the fluorescent nuclear dye SYTO 16. This method has been validated as an indicator of apoptotic cell death [40]. Following a 24-hour exposure to SNP (0.3–3 mM), culture medium was removed and cells were exposed to phenol red-free DMEM medium containing SYTO 16 (2 µM) for 30 min at 37°C. A microplate fluorescence reader (excitation = 485 nm; emission wavelength at 530 nm) was used to automatically quantify the number of apoptotic HaCaT cells by assessing the enhanced SYTO 16 staining.

### Measurement of mitochondrial membrane potential

The mitochondrial inner membrane potential in immortalized HaCaT cells was measured using the fluorescent dye 5,5′,6,6′-tetrachloro-1,1′,3,3′ tetraethylbenzimidazolylcarbocyanine iodide (JC-1), a cationic dye which is used as an indicator of mitochondrial potential in cells [41]. In non-apoptotic cells with intact mitochondria, JC-1 selectively enters the mitochondria where it aggregates and exhibits a fluorescence emission at 590 nm. During loss of mitochondrial membrane potential which features dying or apoptotic cells, JC-1 diffuses throughout the cell and exhibits a green fluorescence emission at 530 nm. Following a 24-hour exposure to SNP (0.3–3 mM), culture medium was removed and cells were exposed to phenol red-free DMEM containing JC-1 (3 µM) for 15 min at 37°C. Red/green fluorescence was measured using a fluorescence plate reader (excitation/emission = 550/600 nm and excitation/emission = 485/535 nm, respectively).

### Measurement of intracellular ROS accumulation

Intracellular ROS levels were estimated in parallel to cell survival using the 2′,7′-dichlorofluorescein diacetate (DCF-DA, 5 µm) fluorescent dye, as previously described [16]. Briefly, the freely cell-permeable DCF-DA was applied to the culture medium at the onset of SNP exposure and is readily converted into 2′,7′-dichlorofluorescein which is then able to interact with intracellular free radicals (primarily H_2_O_2_) to form the fluorescent dye DCF. DCF fluorescence was quantified after 5 hours (excitation = 485 nm, emission = 530 nm) using a fluorescence plate reader.

### Measurement of nitrite formation

Accumulation of nitrite (NO_2_^−^) was measured in culture medium by the Griess reaction using Invitrogen NO colorimetric assay Kit, as described previously [16]. Briefly, cells were exposed to SNP (1–3 mM) and resveratrol (1–30 µM) in DMEM without FBS. Concentrations of nitrite, the end-product of NO production, were quantified 5 hours later by adding 10 µl of Griess reagent (N-(1-naphthyl)ethylenediamine and sulfanilic acid) to 75 µl sample of cell culture medium according to the manufacturer's protocol with minor modifications. The optical density at 548 nm was measured using a micro-plate reader. Nitrite concentrations were calculated by comparison with the optical density of a nitrite solution prepared in culture medium. The detection limit for nitrite measurement in culture medium was 1 µM.

### Measurement of caspases-3 and -9 activities

Caspase-3 and -9 activities were detected using the caspase-3 and -9 colorimetric assay kits, respectively. Briefly, cells were exposed to either vehicle or SNP (0.25–3 mM) alone during 24 hrs or in the presence of resveratrol (1–30 µM). Cells were then lysed and briefly centrifuged (10,000 g). Cytosolic extract was exposed to an assay buffer in the presence of either the caspase-3 (Ac-DEVD-pNA) or capase-9 (Ac-LEHD-CHO) substrate. The optical density at 405 nm was measured 1 hour later with a micro-plate reader. Caspase-3 and -9 concentrations were calculated by comparison with the optical density of a pNA standard prepared in buffer.

### Measurement of p38 MAP kinase activity

The relative amount of p38 MAPK kinase was determined according to manufacturer's protocol using either anti-Phospho-p38 MAPK (Thr180/Tyr182) or anti-p38-MAPK. HaCaT cells were seeded into a 96 well tissue culture plate and exposed for different times to SNP alone or in the presence of resveratrol. Cells were then fixed and incubated with either anti-Phospho-p38 MAPK (Thr180/Tyr182) or anti-p38-MAPK. The wells were then washed, and HRP-conjugated anti-mouse IgG was added to the wells. The wells were washed again and a tetramethylbenzidine (TMB) substrate solution was added to the wells and color developed for 30 min in proportion to the amount of phosphorylated form of p38 MAPK and total p38 MAPK. A stop solution was finally added and changes in the optical density at 450 nm were immediately measured using a micro-plate reader.

### Quantitative real-time RT-PCR analysis

Expression of genes involved in NO signalling pathway was measured using the two-step quantitative real-time human NO signalling pathway RT^2^ profiler™ PCR array (PAHS-062A), according to SABiosciences guidelines. Briefly, cells were exposed to either vehicle or SNP (1 mM) in the presence or absence of resveratrol (10 µM) for 24 hours. Total RNA from each sample was isolated using the RNeasy Mini kit (Qiagen (ONT, Canada). The TURBO™ DNase from (Ambion, Applied Biosystems, ONT, Canada) was then used to remove trace quantities of DNA contamination from RNA samples prior to RT-PCR. Reverse transcription of total RNA was carried out with RT^2^ first strand kit components and cDNA generated was quantified with the RT^2^ real-time PCR SYBR® Green/ROX master using the Applied Biosystems 7500 Real Time PCR System (CA, USA). Six housekeeping genes including β-actin were used to normalize quantification of mRNA target, and non-specific amplifications were verified by dissociation curve. Each value represents the mean of triplicate samples from two independent experiments.

### Statistical analysis

All binding experiments were repeated 3–5 times (each in triplicate) and binding parameters were determined using GraphPad Prism program (version 4.03) (GraphPad Software Inc., San Diego, CA, USA). Survival of vehicle-treated control groups, not exposed to either SNP or various drugs was defined as 100%. A one-way ANOVA followed by Newman Keuls' multiple comparisons or unpaired Student's *t*-tests were used to compare control and treated groups, with p values <0.05 being considered as statistically significant. ΔΔC_t_ based fold-change of genes and bounds of the 95% confidence interval were derived from the average measures and calculated using the RT^2^ Profiler™ PCR Array Data Analysis and compared to gene expression of the control group. A minimal expression change of 3.0 and 0.3 fold had to be reached to be considered as an up-regulated or down-regulated candidate gene respectively.

## Supporting Information

Table S1 Fold change and 95% confidence interval (95% CI) of genes differentially expressed in HaCaT cells treated with resveratrol (RSV, 10 µM alone and SNP (1 mM) alone or in presence of RSV (10 µM). Fold change was compared to gene expression of the control group.
(0.07 MB DOC)
