# Supplementary material for: Protective Action of Resveratrol in Human Skin: Possible Involvement of Specific Receptor Binding Sites
Source: PLoS One. 2010 Sep 23;5(9):e12935. doi: 10.1371/journal.pone.0012935 (PMC2944869; doi:10.1371/journal.pone.0012935)
Supplement: Table S1 — Fold change and 95% confidence interval (95% CI) of genes differentially expressed in HaCaT cells treated with resveratrol (RSV, 10 µM alone and SNP (1 mM) alone or in presence of RSV (10 µM). Fold change was compared to gene expression of the control group. (0.07 MB DOC) [file pone.0012935.s001.doc]

Table S1. Fold change and 95% confidence interval (95% CI) of genes differentially expressed in HaCaT cells treated with resveratrol (RSV, 10 µM alone and SNP (1 mM) alone or in presence of RSV (10 µM). Fold change was compared to gene expression of the control group.

| Genes altered in groups relative to control group  (Representative public ID) | Vehicle + RSV | SNP alone | SNP + RSV |
| --- | --- | --- | --- |
| V-akt murine thymoma viral oncogene homolog 1 (NM_005163) | 8.98  (0.00001, 23.4) | NS | NS |
| Arachidonate 12-lipoxygenase (NM_000697) | 3.17  (0.00001, 8.88) | NS | NS |
| Arginase, type II (NM_001172) | 4.00  (0.00001, 10.3) | NS | NS |
| Catalase (NM_001752) | NS | 0.11  (0.00001, 0.44) | 0.57  (0.00001, 1.45) |
| Copper chaperone for superoxide dismutase (NM_005125) | 4.00  (0.00001, 19.1) | NS | NS |
| Dimethylarginine dimethylaminohydrolase 2 (NM_013974) | 5.66  (0.00001, 15.8) | NS | NS |
| Dual oxidase 1 (NM_175940) | 3.17  (0.00001, 8.77) | NS | NS |
| Dual specificity phosphatase 1 (NM_004417) | 3.56  (0.00001, 9.05) | NS | NS |
| Dynein, light chain, LC8-type 1 (NM_003746) | NS | 0.03  (0.00001, 0.13) | 2.29  (0.00001, 7.46) |
| Epidermal growth factor receptor (erythroblastic leukemia viral (v-erb-b) oncogene homolog, avian) (NM_005228) | NS | 0.05  (0.00001, 0.23) | 0.18  (0.00001, 0.75) |
| Galactosidase, alpha (NM_000169) | 3.17  (0.00001, 9.16) | 3.56  (0.00001, 9.29) | 1.70  (0.00001, 3.66) |
| Glutathione peroxidase 1 (NM_000581) | 3.17  (0.00001, 9.56) | NS | NS |
| Glutathione peroxidase 2 (gastrointestinal) (NM_002083) | 4.00  (0.00001, 11.4) | NS | NS |
| Glutathione peroxidase 4 (phospholipid hydroperoxidase) (NM_002085) | NS | 0.14  (0.00001, 0.44) | 0.38  (0.10, 0.66) |
| Interleukin 8 (NM_000584) | NS | 4.49  (0.00001, 15.9) | 1.84  (0.00001, 4.58) |
| Jun oncogene (NM_002228) | NS | 4.00  (0.00001, 16.1) | 1.38  (0.00001, 4.46) |
| Keratin 1 (NM_006121) | 3.17  (0.00001, 8.00) | 0.35  (0.00001, 0.79) | 0.13  (0.04, 0.22) |
| V-myb myeloblastosis viral oncogene homolog (avian) (NM_005375) | NS | 3.17  (0.00001, 15.9) | 2.11  (0.00001, 8.37) |
| Neutrophil cytosolic factor 1 (NM_000265) | NS | 4.00  (0.00001, 19.4) | 1.74  (0.00001, 6.98) |
| Nitric oxide synthase 1 (neuronal) (NM_000620) | NS | 4.49  (0.00001, 16.30) | 2.33  (0.00001, 5.91) |
| Nitric oxide synthase 3 (endothelial cell) (NM_000603) | NS | 5.04  (0.00001, 20.4) | 2.14  (0.00001, 6.58) |
| NAD(P)H dehydrogenase, quinone 1 (NM_000903) | NS | 4.00  (1.25, 6.75) | 0.69  (0.00001, 2.75) |
| Nudix (nucleoside diphosphate linked moiety X)-type motif 1 (NM_002452) | 4.00  (0.00001, 10.7) | NS | NS |
| Oxidation resistance 1 (NM_181354) | 3.17  (0.00001, 8.00) | NS | NS |
| Polynucleotide kinase 3'-phosphatase (NM_007254) | 5.66  (0.00001, 14.4) | NS | NS |
| Phosphatidylinositol-3,4,5-trisphosphate-dependent Rac exchange factor 1 (NM_020820) | NS | 3.17  (0.00001, 15.4) | 1.55  (0.00001, 6.10) |
| Protein kinase, cAMP-dependent, regulatory, type I, beta (NM_002735) | 4.00  (0.00001, 9.95) | NS | NS |
| Scavenger receptor class A, member 3 (NM_182826) | 4.49  (0.00001, 10.9) | NS | NS |
| Superoxide dismutase 1, soluble (NM_000454) | NS | 0.89  (0.19, 1.59) | 3.25  (0.00001, 9.10) |
| Sulfiredoxin 1 homolog (S. cerevisiae) (NM_080725) | 6.35  (0.00001, 16.2) | 4.49  (0.00001, 14.4) | 2.49  (0.00001, 5.81) |
| Vascular endothelial growth factor A (NM_003376) | 6.35  (0.00001, 17.5) | NS | NS |
| Beta-2-microglobulin (NM_004048) | 0.31  (0.00001, 0.82) | NS | NS |
| Hypoxanthine phosphoribosyltransferase 1 (NM_000194) | NS | 3.17  (0.00001, 7.89) | 2.27  (0.00001, 5.05) |
| Ribosomal protein L13a (NM_012423) | 0.31  (0.00001, 0.79) | NS | NS |
| Actin, beta (NM_001101) | 7.13  (0.00001, 21.4) | 4.00  (0.00001, 8.29) | 2.11  (0.00001, 5.20) |

NS : non significant

Representative public ID obtained from NCBI respectively is provided.
